# Bone Selective Remodeling of Xeno-Hybrid Grafts: A Case Series

**DOI:** 10.3390/jcm14134457

**Published:** 2025-06-23

**Authors:** Roberto Ghiretti, Carlo F. Grottoli, Massimo Molinari, Minh Tam Davide Huynh, Chiara Bonizzi, Claudio Giani, Raffaella De Pace, Giuseppe Perale

**Affiliations:** 1 Private Practice, 46047 Porto Mantovano, Italy; roberto.ghiretti@libero.it; 2 Industrie Biomediche Insubri SA, Via Cantonale 67, 6805 Mezzovico-Vira, Switzerland; carlo.grottoli@ibi-sa.com (C.F.G.); giuseppe@ibi-sa.com (G.P.); 3 de Götzen S.r.l., Acteon Group, 21054 Fagnano Olona, Italy; massimo.molinari@acteongroup.com (M.M.); claudio.giani@acteongroup.com (C.G.); 4 Novartis AG, Campus Novartis, 4056 Basel, Switzerland; tamdavide.huynh@gmail.com; 5 Roche Diagnostics International AG, Via Forrenstrasse 2, 6343 Rotkreuz, Switzerland; chiara.bonizzi@gmail.com; 6 Department of Chemical, Pharmaceutical and Agricultural Sciences, University of Ferrara, 44121 Ferrara, Italy; 7 Faculty of Biomedical Sciences, University of Southern Switzerland (USI), Via G. Buffi 13, 6900 Lugano, Switzerland; 8 Ludwig Boltzmann Institute for Experimental and Clinical Traumatology, Donaueschingenstrasse 13, 1200 Vienna, Austria

**Keywords:** biomaterials, bone grafting, bone regeneration, osteointegration, maxillofacial surgery

## Abstract

**Background:** Maxillofacial bone defects present considerable challenges in oral and reconstructive surgery. While autologous bone grafts are the gold standard, their limitations, such as donor site morbidity and limited availability, have driven the search for alternative biomaterials. SmartBone^®^, a xeno-hybrid graft, offers potential advantages due to its bioactivity and remodeling capacity. **Methods**: This analysis of a series of clinical cases, evaluated the performance of SmartBone^®^ in 10 patients presenting with various maxillofacial bone defects. The patient follow-up period spanned from 2017 to 2019, with a maximum duration of 30 months. Bone grafting was performed, and integration was monitored using Cone-Beam Computed Tomography at multiple timepoints. Bone density changes (ΔCT values) in selected anatomical sites were analyzed to assess graft transformation and integration. **Results**: SmartBone^®^ supported effective bone regeneration and selective remodeling in all cases. One patient required a revision procedure, after which successful integration was observed. Cellular colonization began within weeks, with complete remodeling into mature bone occurring between 6–12 months. Evidence of cortical wall resorption and reformation on the graft’s external surface confirmed this transformation. ΔCT values progressively aligned with native bone densities, indicating structural and functional integration. **Conclusions**: SmartBone^®^ demonstrates strong osteointegrative and site-specific remodeling capabilities, offering a reliable and predictable alternative for maxillofacial bone reconstruction. The study presents several limitations, including the small sample size, inter-patient variability, possible imaging artifacts due to metallic elements in Cone-Beam Computed Tomography scans and the lack of histological confirmation.

## 1. Introduction

Diseases affecting the maxillofacial bone regions represent a significant challenge in surgical practice and often require targeted interventions to restore both function and aesthetics. Among the most adopted therapeutic options, bone grafting procedures play a central role, as they help compensate for bone tissue loss and provide a suitable foundation for potential prosthetic implants. The use of bone grafts is essential to promote the process of osseointegration, which refers to the biological integration between the patient’s bone and the grafted material. After an appropriate healing period of approximately 4–6 months, as reported in the literature [1], this integration enables the correct positioning of dental implants or other prosthetic structures, improving the stability and functionality of the treated area [2]. The primary function of bone grafts is to provide mechanical support and stimulate bone regeneration, with the goal of replacing bone tissue. To achieve these outcomes, graft materials must exhibit four essential biological properties [3]: (i) Osteoconduction, allowing cell migration and vascular infiltration into the scaffold; (ii) Osteoinduction, stimulating mesenchymal stem cells to differentiate into osteoblasts; (iii) Osteogenesis, enabling actual bone formation from viable osteogenic cells; and (iv) Osseointegration, establishing a direct structural and functional connection between living bone and the graft [3]. These requirements are critical for any material intended for effective bone regeneration, especially in high-demand areas such as the oral and maxillofacial region [3].

Radiological examination, particularly CBCT, plays a fundamental role in planning and monitoring maxillofacial surgery procedures, as it provides high-resolution 3D imaging essential for evaluating bone morphology, density and graft integration [4]. It is used to assess the involved evolution of bone tissue, identify the anatomical structures involved, and diagnose any existing pathologies before the intervention. Additionally, it allows for the evaluation of postoperative outcomes, verifying the proper integration of grafted materials or implants [4]. For instance, in bone regeneration or reconstruction procedures, radiographic imaging helps monitor bone volume increase and the healing process, providing essential data for assessing the success of the surgery [4]. However, histological analysis would be the most accurate method to quantify bone growth and transformation, although it is not always possible, particularly after implant placement.

Autologous bone grafts, harvested from the patient’s own body (typically from the iliac crest or mandibular ramus), are considered the gold standard due to their superior biocompatibility and regenerative potential [5,6]. This significantly reduces the risk of rejection [7]. However, autologous bone grafts come with considerable limitations. They are associated with donor site morbidity, including pain, infection, and hematoma [6]; there is a limited volume of harvestable bone, particularly in elderly or medically compromised patients [5,7]; and the requirement of a second surgical site increases operating time, patient discomfort, and overall complication risk [7]. These drawbacks significantly restrict the clinical applicability of autografts, especially in complex or extensive defects [3]. Moreover, the use of non-autologous bone substitutes (e.g., allografts, xenografts, or synthetic grafts) is highly consolidated through routine practice and clinical results from the literature [8,9,10,11], as these grafts can provide mechanical properties, microstructures (e.g., porosity [12]), and compositions that are comparable to those of autologous bone [13,14,15,16]. Bone allografts, from living or cadaveric donors, have been proposed as alternatives, but the risk of disease transmission and the biocompatibility of these grafts continue to pose challenges for their clinical use [17]. For this reason, in recent years, there has been increasing focus on synthetic bone grafts, which are easily available, adaptable to different shapes, osteoconductive, and have predictable absorption rates [18]. Bovine xenografts also prove to be valid supports for bone grafting, due to their similarity to the structure of human cancellous bone [19]. However, the need to sterilize raw animal-derived material could alter its biological and mechanical properties. As a result, low-temperature treatments and composite technologies have been studied to improve the biological and mechanical performance of xenografts, with promising results in both research and clinical settings [20,21]. To overcome these limitations, novel composite biomaterials, such as xeno-hybrid scaffolds, have been developed. These materials combine a bovine-derived mineral matrix with biopolymers and bioactive molecules, enhancing both mechanical performance and biological responsiveness [21].

In general, xenografts are useful for treating defects that require mechanical stability. However, not all biomaterials behave the same, even in equivalent clinical situations [22,23]. Moreover, each biomaterial may require a specific application protocol, which could be invasive depending on the application site. Each clinical case may require a specific biomaterial. Therefore, a thorough understanding of the characteristics and performance of each type of graft, the characteristics of the application site, and the clinical condition of the patient is necessary [22,23]. 

Among modified xenografts, a scaffold composed of processed bovine bone matrix reinforced with biopolymers and active agents has recently been proposed as bone substitute for oral surgery, maxillofacial and dental implantology, and is available as a CE-labeled class III medical device (SmartBone^®^) [21]. This xeno-hybrid material is entitled to have excellent mechanical and bone regeneration properties, proposing to be a great promise for dental and maxillofacial bone tissue engineering [24]. Thanks to its tailored porosity, controlled degradation rate, and structural reinforcement, it promises site-specific remodeling and predictable integration in complex clinical scenarios [24]. Clinical studies have demonstrated that SmartBone^®^ possesses key features required for effective graft performance. It exhibits excellent osteoconductive and osteointegrative behavior, allowing rapid colonization by host cells and progressive replacement with newly formed bone [20,21]. It supports functional remodeling, with the scaffold being resorbed and substituted by bone tissue exhibiting mature lamellar organization and vascularization [21,25]. Notably, SmartBone^®^ has been shown to undergo selective remodeling, where its transformation into cortical or cancellous bone depends on the specific anatomical and mechanical conditions of the recipient site [2,26].

This case series aims to evaluate the clinical performance of SmartBone^®^ in 10 patients affected by diverse maxillofacial bone defects. The primary goal is to assess the material’s osteointegrative behavior, selective remodeling capacity, and safety profile over follow-up periods of up to 30 months. Cone-beam computed tomography (CBCT) combined with dedicated 3D image analysis software was used to monitor changes in bone density and morphology over time. A standardized protocol for image registration and volumetric analysis enabled precise assessment of graft integration, material stability, and the biological response of the host bone tissue. In addition, clinical evaluations were conducted to assess functional and therapeutic outcomes, aiming to confirm the effectiveness of SmartBone^®^ as a reliable and adaptable solution for bone regeneration in a variety of complex maxillofacial pathological scenarios.

## 2. Materials and Methods

### 2.1. Patients Selection

This case series analysis included 10 adult patients (age > 18 years) presenting with localized maxillofacial bone defects requiring grafting procedures. Patients were recruited from a private dental practice in Porto Mantovano (Italy). Inclusion criteria required the presence of a bone defect in the maxillofacial region suitable for SmartBone^®^ grafting (Industrie Biomediche Insubri SA, Mezzovico-Vira, Switzerland). Exclusion criteria involved patients with 3D CBCT clinical exams not acquired using the X-MIND Trium device, or those not meeting the specific parameters described in the radiological analysis method. Additionally, patients treated with biomaterials other than SmartBone^®^, clinical cases with follow-up periods shorter than one year, and cases where the defect anatomy did not allow clear discretization of the initial parameters were excluded. Based on the clinical indication and anatomical requirements, 4 patients received SmartBone^®^ granules (0.25–1 mm), 3 patients were treated with preformed standard plates (3 × 25 × 15 mm), and 3 received custom-made On-Demand grafts obtained via CAD/CAM processing.

The total follow-up duration varied among patients and extended up to 30 months. CBCT scans were performed at multiple timepoints to monitor bone regeneration and remodeling.

This study was conducted in compliance with the Declaration of Helsinki, as revised in Fortaleza (2013), and adhered to the principles of Good Clinical Practice and the ISO 14155 standard for clinical investigations. All patients signed a written informed consent form after receiving detailed information about the study objectives, procedures, and data use. The graft material used, SmartBone^®^, is a CE-marked Class III medical device. The clinical procedures were performed as part of standard post-marketing clinical follow-up in accordance with ISO 13485:2016 [27]. Based on this regulatory framework, and since no investigational use of the product was performed, the study did not require specific approval from an ethics committee or institutional review board.

### 2.2. Analysis Design

SmartBone^®^ bone grafts was used in the treatment procedure and these was supplied in three variants: (1) microchips of size 0.25–1 mm, (2) plates of size 3 × 25 × 15 mm, or (3) On-Demand SmartBone^®^ blocks which is computer numerical controlled (CNCed) to fill the defect reconstructed from computer tomography (CT scans). This is a computer-aided design and computer-aided manufacturing (CAD/CAM) solution to accurately replicate the patient defect geometry. All the SmartBone^®^ grafts were used as produced, unless otherwise specified. The applied radiological method involved 3D Cone Beam Computed Tomography (CBCT) exams acquired with an X-MIND trium system (de Götzen, Acteon Group, Fagnano Olona, Italy) by using different scanning protocols defined by: image sizes (FOVs: 40 × 40/60 × 60/80 × 80/110 × 80 mm), resolutions (voxel spacing: 0.1/0.15/0.2/0.3 mm), loading factors (Tube Voltage: 85/90 kV, Anodic Current: 8/10 mA, Exposure time: 6/7.2/9 s, etc.) and follow-up acquisition time. Image processing and analysis were performed using the open-source software 3D Slicer v4.10.2 (www.slicer.org), a platform for medical image computing and 3D visualization. The following modules were used: “Transforms” for manual registration, “Crop Volume” for image cropping, and “Elastix” (SlicerElastix extension) for automatic rigid registration. In addition, a custom Python (version 3.8.10) script using the Insight Segmentation and Registration Toolkit (ITK, v5.2) was developed to compute voxel-wise distances and to extract CBCT values at selected anatomical landmarks [28,29]. The procedure initially involved the registration of images, acquired at various post-operative times and manually aligned with the pre-operative image. This was followed by automatic registration. In this phase, it was assumed that the maxillary region was a rigid body, allowing only translations and rotations (rigid transformations), excluding elastic deformations that could distort the anatomical data. Subsequently, a “cropping” operation was performed to exclude the bone portion not involved in the procedure, to focus the automatic registration only on the treated area. Once the “cropping” was performed, rigid automatic registration was applied using the “Elastic” module of the 3DSlicer software, refining the manual alignment. The accuracy of the registration was measured by observing a stable anatomical structure common to all of the patient’s images, such as the lateral upper bone areas of the jaw. The distances between the surfaces of the images were calculated both qualitatively, generating a colour map to visualize alignment differences, and quantitatively, by calculating point-to-point distances along the three-dimensional axes. Regarding the evaluation of bone density, the corresponding CBCT values were measured at various anatomical sites. The differences in CBCT values between treated and untreated cortical and trabecular areas were monitored over time to observe the evolution of bone remodeling. Hereafter, the semi-automatic image processing protocol defined for intra-patient analysis and evaluation is reported. All the following steps were performed for each patient across all his/her available subsequent *D CBCT acquisitions, which were ordered temporally, from time −1 = pre-surgery, then time 0 = surgery, follow-up up to time 3 = 3 months after Time 0, and so on.

### 2.3. Multistep Images Registration

#### 2.3.1. Manual Alignment (Step One)

The first step involved the manual alignment of all subsequent scans with respect to the baseline acquisition (Time −1, pre-surgery). The goal was to minimize the distances between the regions of interest (ROI) (i.e., the site which underwent bone grafting surgery, which might have been a minor or major part of all the imaged volume) which ensured a proper starting point for the following automatic registration step described below [30,31,32]. At first, the hypothesis of the maxillo-facial bone region as a non-deformable body was made. Only translations and rotations were allowed, because only rigid transformations were considered in this protocol. Although this assumption might be true for most of the non-growing population (to which the enrolled patients belonged to), this hypothesis could not be considered absolute since the bone architecture and its remodelling process were heavily affected by external loading and physiological process that might have occurred in a time of 2–3 years, such as for the patients considered in this study. In turn, external loading was dependent on (partial) edentulism, presence of orthodontic treatments, dental implants, etc., characteristics that belong to most of the patients in this study. On top of all these conditions, the cases considered in this study shared some kind of bone pathologies which were treated with bone grafting, adding another major factor in bone remodelling [33]. Thus, some sort of deformation of the anatomic structures, which were supposed to be stable and rigid, was observed (e.g., relative movement of teeth, bone reabsorption, etc.). Furthermore, errors in the patient’s setup (such as head orientation or inclination during scan) contributed to the increase in the variability of the localization and orientation of the supposedly rigid structures. The proposed protocol aimed to overcome the factors that could lead to the failure of rigid registration: it did not use elastic transformations to promote the maintaining of the real anatomical structures and it avoided any mathematical process that might have created unreal deformations. In conclusion, it was preferable to accept an error in the rigid registration due to physiological deformations rather than an error induced by using an elastic deformation that altered the informative content of the imaged volumes. In this first phase of manual alignment, the minimum manual translation step needed was 1 mm and the minimum manual rotation step needed was 1 degree. It had just to be a gross, manual overlapping of the volumes.

#### 2.3.2. Cropping of the Region of Interest (Step Two)

The second step involved cropping the scan to isolate the maxilla (upper or lower) containing the area of interest [34,35,36]. It was decided to exclude the bone portion not involved in the treatment to optimize the following automatic registration only for the area of interest; indeed, maxilla and mandible could have different relative position throughout the acquisitions depending on the position of the temporomandibular joint (TMJ) which could invalidate the automatic registration. The other reason why this crop was applied was because the points of the virtual specimens (or point of observation) to be acquired in the following steps belonged only to the treated maxilla portion. Moreover, since the acquired volumes over time were not of the same size and/or did not image the same patient’s region, the volume cropped was also limited by the minimum common volume imaged by all acquisitions.

#### 2.3.3. Automatic Rigid Registration (Step Three)

In the final step, a rigid automatic registration of all subsequent (cropped) volumes was performed with respect to the baseline (Time −1) scan [27,30,36,37]. 3D Slicer’s Elastic module implemented a voxel-based automatic rigid registration which was used to fine-tune the manual registration [38]. This registration was applied to the cropped volumes obtained as output of the previous step. The automatic registration was performed using the “Elastix” module, integrated in 3D Slicer v4.10.2, with 250 optimization steps per iteration across four iterations; registration was constrained to rigid transformations only.

### 2.4. Metrics

The accuracy of the registration process was evaluated by the detection of a stable anatomic structure common to all the cropped volumes of the examined patient. This stable region should not have included teeth if any of them underwent any endodontic treatment extraction or implant application during the examination period. Moreover, this region should not have been impacted by any surgical intervention or major anatomical deformation. As a result, most often this region included only maxillary lateral bone areas. It is worth noting that this region was case-specific. Once the region has been defined, it was segmented for every exam to define the external surface (any physiological hole such as the mental foramen within the volume was not considered but filled). Finally, for all exams from Time 0 and ahead, the computation of the surface distance with respect to the pre-surgery stage (time −1) was computed.

Qualitatively, a colour map volume which summarises the overall distances of the two surface meshes was rendered and visualised [12,31,34,35,39,40]. This process allowed the user to recognise if the volumes might have benefited from a second iteration of the manual and automatic registration procedure. 

Quantitatively, point-to-point distances of the meshes were computed along the three-dimensional axes using the voxel spacing as a unit of measure (e.g., point B on surface 2 was distant 1 voxel from corresponding point A on surface 1). A custom Python (version 3.8.10) plugin was developed to compute point-to-point mesh distances, a functionality not available in 3D Slicer v4.10.2. For the clinical application of this study, the volumes registration was considered successful if at least 95% of the distances were below 1 voxel error size and if 98% of the distances were sub-millimetric.

### 2.5. Bone Density Evaluation via Relative CBCT Radiopacity

The bone density was evaluated in several anatomical sites by means of relative measures and temporal trend of the cone beam computed tomography (CBCT) values. It should be noted that CBCT does not allow absolute quantification of bone mineral density; instead, the grey values were used as relative indicators of radiopacity changes over time. Therefore, the analysis focused on comparing the radiographic appearance between regions and over different timepoints, rather than reporting absolute density values. The assessment is summarized in the following phases:

(a) Patient-specific manual selection of pre-defined bone landmarks on the pre-surgery stage (time −1) cropped volume: buccal and lingual cortical bone, trabecular bone in treated and healthy region. The types of landmarks were decided beforehand and, since the volumes were registered, their coordinates should have remained constant in all the acquisitions. As reported above, the cropped volumes included only one of the two maxillae, the one interested by the treatment. This was reasonable since the comparison of healthy and treated cortical and trabecular regions should have been carried out only within the same maxilla. Using the landmark coordinates (defined as single voxels), CBCT values were extracted for each timepoint using a custom Python tool built upon the SimpleITK and NumPy libraries. For more robustness, each landmark had a multiplicity of three and its CBCT value was averaged with the values from adjacent voxels (within a *n × n* cubic probe centred in the selected landmark).

(b) Patient-specific evaluation of the “variable” landmark. There were two kinds of variability: intra and inter patient. Due to inter-patient variability, since our dataset included different types of surgical cases, a patient-specific approach was adopted. This required selecting anatomical landmarks differently for each patient, depending on the surgical site and individual anatomy. Intra-patient variability was due to the bone remodelling process and the assimilation of the osteosynthesis material, which, as a result, might have varied the position and conformation of the bone (particularly and most often, of the cortical bone). Consequently, there was a landmark which might not always have represented the same structure in each following exam (e.g., a buccal cortical bone which advanced and then receded over time, due to the addition and assimilation of the osteosynthesis material, respectively). Therefore, for acquisitions of the same patient, this “moving” landmark had to be repositioned every time.

(c) Computation of the differences between CBCT values of the same acquisition (buccal vs. lingual cortical bone CBCT values, healthy vs. treated trabecular bone CBCT values, fixed (i.e., buccal) vs. moving (i.e., lingual) cortical bone CBCT values) and observation of the evolution of these differences over time (i.e., for all the exams). Given the known limitations of CBCT in providing standardized density data [41], these differences were interpreted as indicators of relative radiopacity variation. CBCT values of different acquisitions (even with the same machine, patient and load factors) were dependent on the intrinsic variance of the CBCT systems. Thus, to compare the bone density in different volumes, an evaluation of the evolution of the differences between reference anatomical regions was made.

(d) Since the variance of CBCT grey values was due to the intrinsic variance of the X-ray systems and to the image artefacts caused by the metal implanted into the patient’s mouth during the healing process, further analysis could be performed. In particular, patients were divided into two subgroups: (i) those affected by significant metal-induced artefacts and (ii) those with negligible artefacts. For the first group, quantitative analysis of CBCT values was considered unreliable and thus excluded from the quantitative evaluation of bone regeneration. In these cases, only qualitative assessments based on visual inspection and surface mesh comparison were performed. For the second group, i.e., cases with low or no artefact contamination, temporal changes in CBCT values were systematically analyzed, providing both quantitative and qualitative evidence of the bone regeneration process. The identification and exclusion of artefact-affected regions were performed visually by expert radiologists, and volumes exhibiting severe streak artefacts in the regions of interest were considered non-evaluable for densitometric assessment. Further analyses that could be performed to improve the management of metal artefacts include the following: the application of artefact-reduction algorithms based on iterative reconstruction or spatial filtering methods; automatic or semi-automatic segmentation to exclude contaminating regions from density analysis; the use of calibration techniques to compensate for artefact-induced variations; or the employment of alternative CBCT scanning protocols optimized to reduce metal artefact effects. However, these techniques were not implemented in the present study and remain prospects for future developments.

### 2.6. Quantification of Bone Regeneration

The aim of this work was to demonstrate how the xeno-hybrid bone graft material SmartBone^®^ promotes bone regeneration and undergoes selective remodelling. Regeneration process can be observed thought the observation of the zone where the graft was inserted over time. In particular, the observational process focuses on measurement of the delta CT values trend between buccal and lingual cortical, and between treated and non-treated medullar. These are the formulas to calculate the delta CT values:

$$\Delta_{1}={\bar{CT}}_{lingual\;cortical}-{\bar{CT}}_{buccal\;cortical}$$

$$\Delta_{2}={\bar{CT}}_{treated\;medullar}-{\bar{CT}}_{non-treated\;medullar}$$

**Figure 1:**
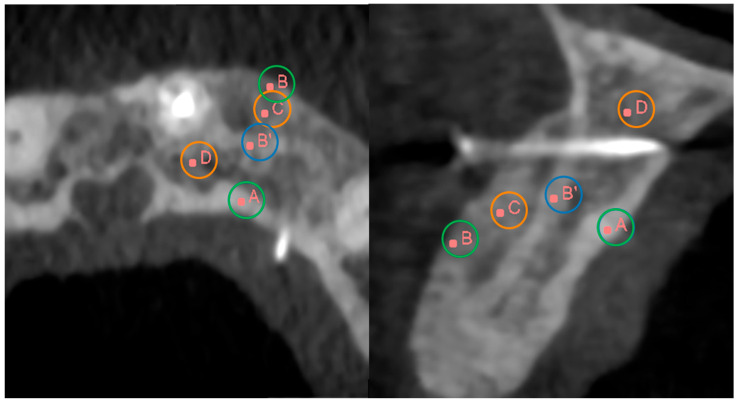
Axial (**left**) and sagittal (**right**) CBCT views of the maxillary bone. Scan images highlight key anatomical landmarks used to analyze bone regeneration after SmartBone^®^ grafting. Points of interest include: (A) lingual cortical, (B) new buccal cortical, (B’) original buccal cortical, (C) treated medullary, and (D) non-treated medullary. These points are used to calculate ΔCT values, assessing cortical and medullary remodeling over time.

The calculus of these delta values was based on the density of the bone in different points which were voxel values in significant anatomical landmarks (Figure 1). Specifically, these areas of interest were as follows:

A: Lingual cortical;
B: New buccal cortical;
B’: Original buccal cortical;
C: Treated medullar;
D: Non-treated medullar.

In view of this the new formulas were:

$$\Delta_{mobile\_cortical}={\bar{CT}}_{lingual\;cortical}-{\bar{CT}}_{buccal\;cortical}=\bar{A}-\bar{B}$$

$$\Delta_{fixed\_cortical}={\bar{CT}}_{lingual\;cortical}-{\bar{CT}}_{buccal\;cortical}=\bar{A}-\bar{B^{\prime }}$$

$$\Delta_{medullar}={\bar{CT}}_{non-treated\;medullar}-{\bar{CT}}_{treated\;medullar}=\bar{D}-\bar{C}$$

## 3. Results

A total of 10 clinical cases were treated with the SmartBone^®^ bone graft in either granular or plate form. Of these 10 cases, 9 were considered successful, 1 was partwise successful (case 7) but did not require revision. In all cases, SmartBone^®^ graft demonstrated osteointegrative properties, and the ability to remodel upon need. The remodelling seemed to start immediately after surgery and continue for 6–12 months. The remodelling of the graft into new bone was evident from the resorption of the original distal cortical wall and the formation of a new distal cortical wall. This was particularly evident in case 2, where a bone reconstruction procedure was performed to address bone resorption around a prosthetic arch in the upper jaw. After 3 months, a thicker jaw and initial remodeling were observed. At 6 months, the original cortical wall had completely remodeled into cancellous bone, and a new cortical wall had formed at the edge of the graft.

### 3.1. Cases Description:

#### 3.1.1. Case 1—PZ001

**Figure 2:**
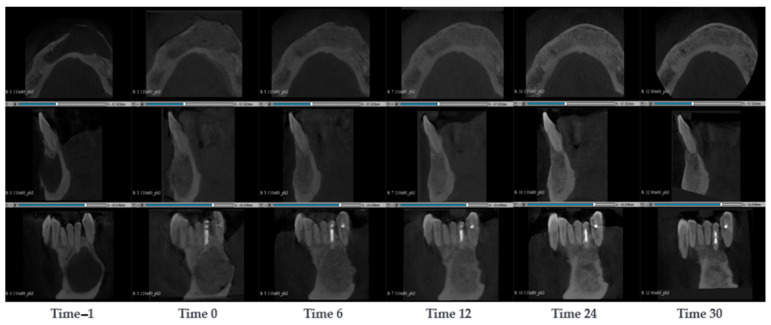
CBCT images of a 69-year-old male with a large mandibular cyst. The cyst was removed and SmartBone^®^ microchips were inserted. The images show the progression of bone healing over time, from pre-surgery (Time −1) to 30 months post-surgery (Time 30).

A 69-year-old male presented with a large mandibular cyst, characterized by a continuous lingual cortical layer and a very thinned vestibular cortical layer. During the surgery, the cyst was removed and SmartBone^®^ microchips 0.25–1 mm were inserted mixed with concentrated growth factors (CGFs; Medifuge MF200, Silfradent S.R.C., 47018 Santa Sofia FC, Italy) using a special technique that ensured a product richer in regeneration factors and easy to manipulate [2]. Seven CBCT scans were evaluated: before the surgery, immediately post-surgery and after 6, 12, 24 and 30 months. CBCT images (Figure 2) show the vestibular cortical layer with few open points which appeared even bigger than in the previous exam (Time −1). The grafted material appeared with light grey and the distribution was a bit irregular with blank spaces. After 6 months the images showed a better density uniformity and the colour in the grafting region turned out to be grey, like the colour of the mandibular native bone. This change was due to the mineralization and transformation of the grafted biomaterial, since the biomaterial is homogeneous, which allows for a more consistent remodeling process. After 12 months, the increment of bone mineralization appeared more evident, and it started to be a noticeable differentiation of the bone quality with a visible thin cortical external layer. After 24 months the external cortical layer was clear and distinguishable on all the external vestibular cortical sides where the original cortical layer was present. After 30 months, the distal cortical wall appeared well-formed and visible, with a constant thickness over the entire distal surface (where the original cyst was placed). Compared to the previous exams (24 months), no relevant differences in terms of bone regeneration were noticeable. The patient shows complete regeneration of the surgical zone, with a complex and articulated structure, which corresponds to the newly formed external cortical layer. This result highlights the capacity of SmartBone^®^’s structure to differentiate.

#### 3.1.2. Case 2—PZ002

**Figure 3:**
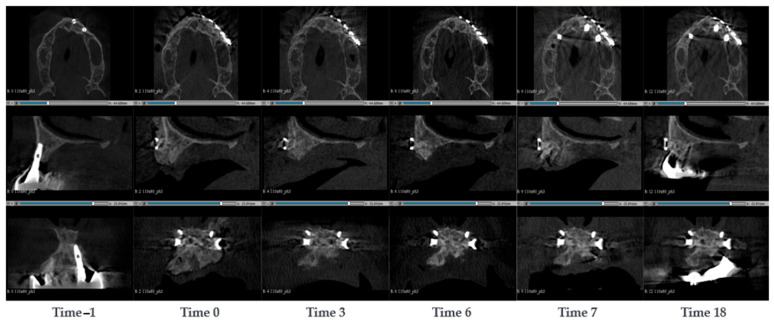
CBCT images of a 54-year-old woman with bone resorption around a prosthetic arch in the maxilla. The defect was repaired by a fencing procedure, filled with SmartBone^®^ microchips. The images show the progression of bone healing over time, from pre-surgery (Time −1) to 18 months post-surgery (Time 18).

A 54-year-old woman presented with bone resorption around a prosthetic arch in the maxilla. The defect was repaired by a fencing procedure, where it was filled with SmartBone^®^ microchips 0.25–1 mm and covered with a resorbable collagen membrane (PARASORB RESODONT^®^, RESORBA Medical GmbH, Nurnberg, Germany). After 7 months, new dental implants were successfully inserted. CBCT scans were conducted at the timepoints −1, 0, 3, 6, 7, and 18 months after the surgery (Figure 3). As the patient had a pre-existing dental arch implanted, the image at time 0 was noisy. However, it could be observed that bone was resorbed around the dental implant. The bone resorption was particularly evident in the alveolar. At the surgery stage, the dental arch was removed, the alveolars were filled and the bone was built up with SmartBone^®^ microchips. To keep the microchips in place, a fence was used. The fence was attached with four screws, one in each corner, and other four were used to attach the membrane. After 3 months, a larger jaw thickness could be observed and that the original cortical started resorbing, which could be interpreted as an initial sign of remodelling (Figure 3). After 6 months, while the original cortical wall was fully remodelled into cancellous bone, a new cortical wall was formed at the edge of the grafting material. Since SmartBone^®^ exhibited osteointegration and it was remodelled into native bone tissue, it was feasible to reinsert a new dental arch after 7 months. In addition, a dental implant was inserted in the left side maxillary premolar, although this area was not treated with SmartBone^®^. After 18 months, the regenerated bone tissue still displayed integrity. The maxilla thickness remained stable, and the cortical wall was stable underneath the fence.

#### 3.1.3. Case 3—PZ003

**Figure 4:**
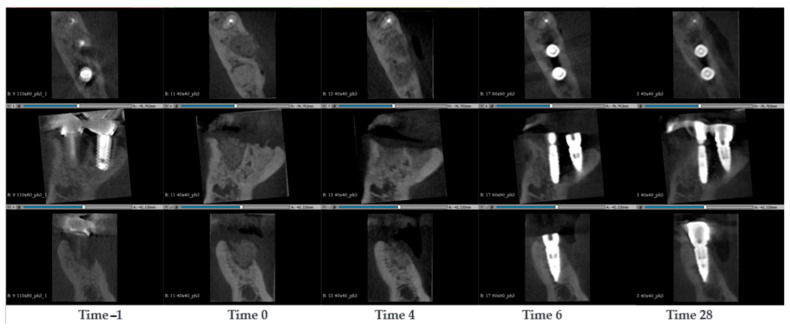
CBCT images of a 59-year-old woman with bone resorption between two dental implants. The implants were removed, and the alveolar bone void was filled with SmartBone^®^ microchips. The images show the progression of bone healing over time, from pre-surgery (Time −1) to 28 months post-surgery (Time 28).

A 59-year-old woman presented with bone resorption between two dental implants (position 36 and position 35), requiring socket preservation and the implant in position 35 seemed to have periodontist [2]. The dental implants were removed displaying good jawbone except for alveolar bone void. The void was filled with SmartBone^®^ microchips 0.25–1 mm. Six months later, the bone recovered and remodelled; thereafter, two new dental implants were inserted. CBCT scans were conducted at the timepoints −1, 0, 4, 6 and 28 months after surgery (Figure 4). At the pre-surgery stage (Time −1), major bone resorption around the dental implant was observed, while the distal cortical wall was partwise missing. At time 0, it was observed that the SmartBone^®^ microchips filled the void of the dental implant (position 35) and the void of the defect. There was an obvious x-ray absorption difference between the chips filled region (dark grey) and the native bone (light grey) (Figure 4). After 4 months, the void was still filled with bone which resulted in being more similar in colour to the native bone. Moreover, it was observed that the distal cortical wall started to recover, suggesting that the osteogenic cells could successfully remodel SmartBone^®^ microchips. After 6 months the dental implants were re-inserted, and they remained stable after 28 months, indicating a successful surgery. At the same timepoint it was also observed that the bone around the implant maintained its integrity. The cortical wall remained thick and strong, although this was difficult to observe due to the image artefacts caused by the dental implants.

#### 3.1.4. Case 4—PZ006

**Figure 5:**
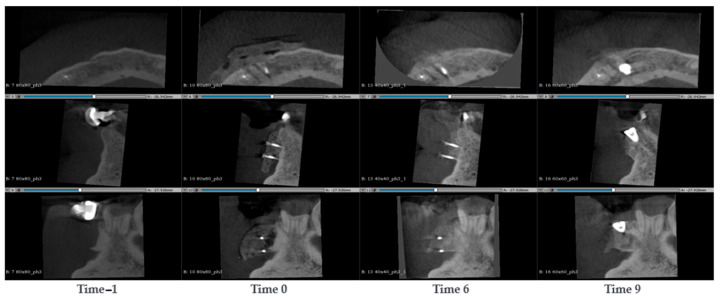
CBCT images of a 59-year-old man with bone resorption around a missing tooth, replaced with a bridge. To recover the bone height, a SmartBone^®^ plate was implanted. The images show the progression of bone healing over time, from pre-surgery (Time −1) to 9 months post-surgery (Time 9).

A 59-year-old man presented with bone resorption around a missing tooth at position 43, that was replaced with a bridge. Underneath the bridge, the bone resorbed but still had a thick cortical wall. It was attempted to recover the height by implanting a SmartBone^®^ plate 3 × 25 × 15 mm which was fixed with two nails. CBCT scans were conducted at the timepoints −1, 0, 6 and 9 months after the surgery (Figure 5). After 6 months, the cortical wall in contact with the SmartBone^®^ plate started to disappear, and the qualitative difference between native bone and graft is very small. A uniform cancellous structure grew up from the original native bone to the SmartBone^®^ plate. No new cortical wall has been formed at this point. After 9 months, a new crown was implanted, which introduced more noise in the radiology images. Following this action, a cortical wall started to form in the lower parts of the SmartBone^®^ plate.

#### 3.1.5. Case 5—PZ009

**Figure 6:**
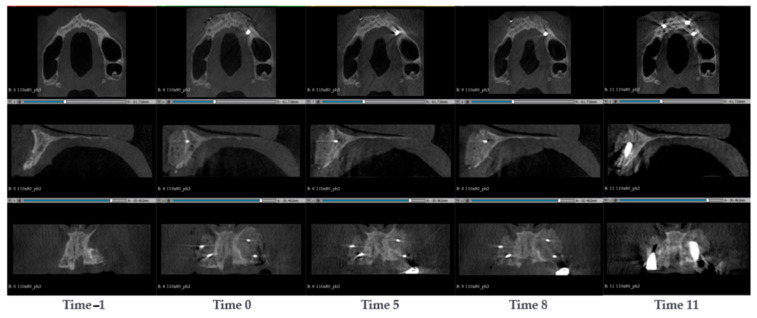
CBCT images of a 57-year-old man presented thin maxillary bone with deep groves caused by cavities in the root of the missing teeth. Two SmartBone^®^ plates were fixed in the region of the incisors and canine. The images show the progression of bone healing over time, from pre-surgery (Time −1) to 11 months post-surgery (Time 11).

A 57-year-old man presented with a thin maxillary bone with deep groves caused by cavities in the root of the missing teeth. More specifically, in the maxilla, only the following were present: the tooth at position 11 devitalized and with a broken crown, the tooth at position 13 devitalized with a deep inflammation, and the adjacent bone was absent. Two SmartBone^®^ plates 3 × 25 × 15 mm were fixed in the region of the incisors and canine, one on the right and one on the left, each secured with two pins. 5 CBCT images at times −1, 0, 5, 8 and 10 were used to characterize the defect and the bone regeneration (Figure 6). During the surgery intervention, a well-defined original cortical was observed. After 5 months, the original cortical was still clearly visible, and there was no clear formation of a new distal cortical wall. After 8 months, the colour of the bone graft started to look like that of the native bone. The resorption of the original cortical wall was clear (particularly on patient’s left side), and signs of a new distal cortical wall formation were observed. After 11 months, two implants were inserted at position 12 and 22. The introduction of these implants introduced artefacts to the images, which might explain why it was no longer feasible to observe the new distal cortical wall.

#### 3.1.6. Case 6—PZ010

**Figure 7:**
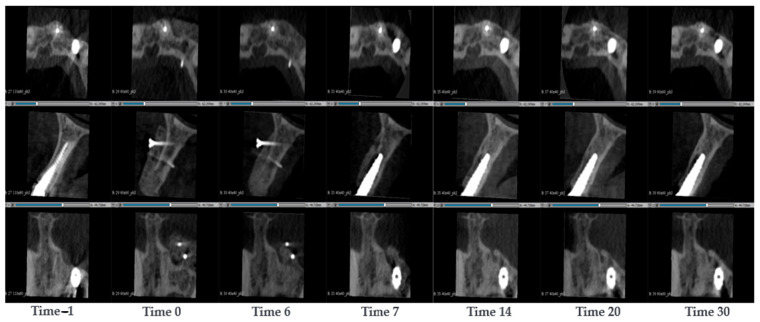
CBCT images of a 44-year-old woman treated for a thinning of the left maxillary, threatening the integrity of a dental implant. The implant was removed and a SmartBone^®^ plate was implanted to recover the maxillary thickness. The images show the progression of bone healing over time, from pre-surgery (Time −1) to 11 months post-surgery (Time 30).

A 44-year-old woman was treated for a thinning of the left maxillary, threatening the integrity of a dental implant at position 22. The implant was removed and a SmartBone^®^ plate was implanted to recover the maxillary thickness. CBCT scans were conducted at the timepoints −1, 0, 6, 7, 14, 20 and 30 months after the surgery (Figure 7). In the pre-surgical image taken one week before the surgery, a loss of volume in the cancellous alveolar bone was observed. Moreover, the dental implant in position 22 seemed to be implanted in a too high position, due to the loss of volume, this was almost protruding the distal cortical surface. During the surgery, the implant in position 22 was removed and a SmartBone^®^ plate 3 × 25 × 15 mm was inserted. The plate was attached with two nails, and it seemed like one of them penetrated the inner cortical wall. After 6 months, the original cortical wall started to resorb and there were patterns of a new cortical wall formation. In the CBCT taken after 7 months the graft has undergone volumetric resorption, and it appeared thinner than at time 0. In consideration of this positive effect, the surgeons inserted a new dental implant. The remodelling was followed up with at 14, 20 and 30 months after surgery. “The general trend observed was that the plate progressively decreased in thickness over time and was remodeled into a more defined and structured cortical wall. A time-dependent selective remodeling of the biomaterial was evident, leading to the formation of both a new cortical layer and underlying cancellous (spongious) bone. While an overall increase in volume was observed, the biomechanical forces acted to reshape only the necessary portion of the graft, remodeling the functional area while the excess material was gradually resorbed. Notably, even after 30 months, no signs of the implant protruding beyond the cortical boundary were detected.

#### 3.1.7. Case 7—PZ011

**Figure 8:**
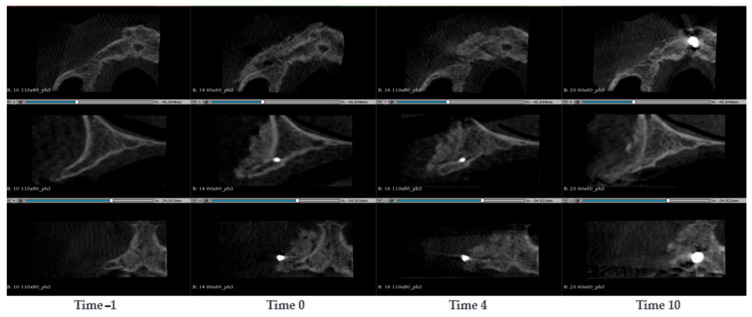
CBCT images of a 73-year-old woman with multiple devitalized teeth and bone resorption in the maxilla. A large SmartBone^®^ plate was fixed to the right-side maxilla. The images show the progression of bone healing over time, from pre-surgery (Time −1) to 10 months post-surgery (Time 10).

73-year-old woman presented multiple devitalized teeth and bone resorption in the maxilla. A large SmartBone^®^ plate was fixed to the right-side maxilla between the teeth at positions 11 and 17, aiming at regenerating the bone thickness so that a tooth implant could be inserted after 10 months. CBCT scans were conducted at the timepoints −1, 0, 4 and 10 months after the surgery (Figure 8). At time −1 (before surgery), it was observed that only teeth at positions 11, 17, 21, 22, 23, and 27 were present, with devitalization observed in teeth 11, 21, 22, and 23. Encapsulation affected all remaining teeth, while teeth 24, 25, and 26 consisted only of a metal crown supported by a bridge. Significant jawbone thinning was noted between teeth 11 and 17 (right-side maxillary), and near teeth 24, 25 and 26. After 4 months, there were signs of failure as the posterior nail was no longer visible, and it seemed to have come loose with a section of the SmartBone^®^ plate. Anyway, in the region where the SmartBone^®^ plate was still present, the original distal cortical wall appeared to start resorbing. After 10 months, the nail that attached the plate was removed such that a dental implant could be inserted at position 11. Following the removal of the nail, more material was lost, indicating poor osteointegration. Some remnants of the material were detectable, most in the upper area, but compared to previous examinations it was greatly reduced in volume. This was noticeable at the top, where some osteosynthesis material remained, a jagged double-front of distal cortical, a sign that the new one was forming and reducing the original one.

#### 3.1.8. Case 8—PZ013

**Figure 9:**
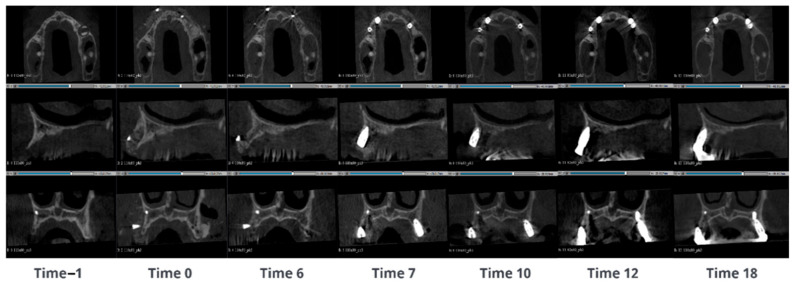
CBCT images of a 58-year-old woman with devitalization of multiple teeth and bone resorption in the maxillary. SmartBone^®^ On-Demand plates were inserted on both sides of the front maxillary. The images show the progression of bone healing over time, from pre-surgery (Time −1) to 18 months post-surgery (Time 18).

A 58-year-old woman presented with multiple indications, including devitalization of multiple teeth and bone resorption in the maxillary. Two SmartBone^®^ On-Demand plates were inserted on both sides of the front maxillary. This allowed three dental implants planned to be inserted after 7 months and another one after 12 months. CBCT scans were conducted at the timepoints −1, 0, 6, 7 and 10 months after the surgery (Figure 9). From the CBCT images at surgery stage the distal cortical was still well distinguishable from the material. Some of the nails appeared to have also pierced the proximal cortical of the maxillary bone. After 6 months the graft plates, especially on the upper right arch, were absorbed by the maxillary bone, significantly increasing its thickness. The distal cortical was no longer clearly visible, while the material took on a shade of colour very similar to the patient’s natural bone. At time 7, three implants were inserted approximately near teeth at position 13, 14 and 24, respectively. The nails were removed. The right maxillary bone was thickened compared to the status at pre-surgery and had a new distal cortical formed by the plate. On the upper left side, however, it seemed that the plate was struggling to be absorbed, particularly near tooth at position 22, where the bone remained as thin as at the pre-surgery stage. It seemed that the plates did not perfectly adhere to the cortical, preventing a correct process of osteoregeneration. Despite this, the plate on the left side took on a colour shade similar to the natural bone but still did not present a cortical formation. After 7 months, overdenture implants were positioned. The plate on left side was not well integrated, and the surgeon did not insert the implant in position 22. At time 10, the upper right arch had a good distal cortical, well defined and of satisfactory thickness. On the upper left arch, instead, it was observed that the plate did not fully adhere to the natural bone. The distal cortical in this case remained fairly as the original one. After 12 months, it was inserted an implant near tooth at position 25. While on the right side the plate was increasingly integrated and the new cortical more and more evident, on the left side the plate remained in place but was not integrated. The overall volume of the left plate appeared qualitatively greater compared to the plate on the right. After 18 months, the left plate was still visible but separated from the native bone while the right plate was integrated into the bone. The left plate was still visibly separated from the original bone, which had a well-defined distal cortical, although the bone thickness was thin for the teeth at position 22 and 23, respectively. The right plate was almost completely absorbed by the bone and was almost no longer visible, the distal cortical was well defined and of a shade similar to the natural one. No major/significant changes were detected compared to the previous examination.

#### 3.1.9. Case 9—PZ014

**Figure 10:**
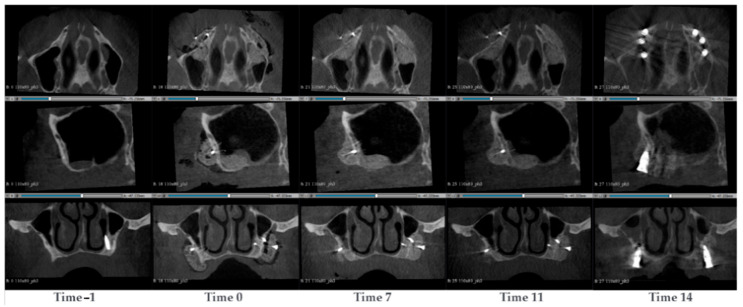
CBCT images of a 63-year-old man with atrophy in the maxillary sinus. SmartBone^®^ On-Demand plates were inserted in the left and right maxillary to recover the bone. The images show the progression of bone healing over time, from pre-surgery (Time −1) to 14 months post-surgery (Time 14).

A 63-year-old man presented with atrophy in the maxillary sinus. Two SmartBone^®^ On-Demand plates were inserted in the left and right maxillary to recover the bone. This allowed a total of 6 new dental prothesis, 3 on each side, planned to be inserted after 12 months. CBCT scans were conducted at the timepoints −1, 0, 7, 11 and 14 months after the surgery (Figure 10). Prior to the surgery, it was observed that the maxillary bone was mostly absent. Additionally, a dental prothesis was present, but part of it was protruded through the maxillary. After the surgery, a clear contrast difference between the SmartBone^®^ plate and the native bone could be observed. After 7 months, the material started to absorb, the natural distal cortical started to thin and merge with the inserted material. A new distal cortical front was not yet present. At time 11, the material was increasingly acquiring a shade of colour similar to the bone, and the natural cortical almost completely disappeared. A new distal cortical front, however, was not present. At the same time, six implants were inserted. After 14 months, a new distal cortical was observed.

#### 3.1.10. Case 10—PZ015

**Figure 11:**
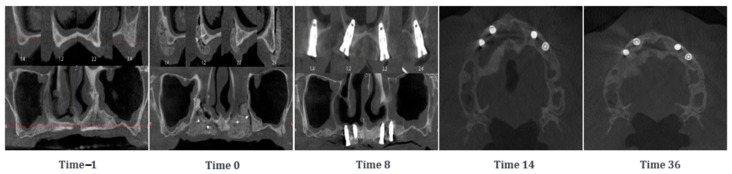
CBCT images of a 64-year-old woman with atrophy in the maxillary bone. SmartBone^®^ On-Demand plates were inserted, covering both the right and left alveolar processes of the upper arch. The images show the progression of bone healing over time, from pre-surgery (Time −1) to 36 months post-surgery (Time 36).

A 64-year-old woman presented with a severely atrophic and thin maxillary bone, completely edentulous in the upper arch. Two SmartBone^®^ On-Demand plates were placed bilaterally, secured with two screw each, covering the alveolar processes. CBCT scans were conducted at the timepoints −1, 0, 8, 14 and 36 months after the surgery (Figure 11). Initial postoperative CBCT images showed that the original cortical bone was still clearly visible and well distinct from the plates. At 8 months, the distal cortical bone in treated areas had largely resorbed, and the residual material blended in colour with the untreated native bone. An increase in maxillary bone volume was qualitatively observed, particularly on the left side. The fixation screws were removed, and four implants were placed at positions 12, 14, 23/24, and 25/26. At 14 months, implants were loaded with a bar-supported overdenture. Imaging showed that the left side maintained some well-integrated material with a clearly appreciable new distal cortical wall, while on the right side, the material appeared almost completely resorbed. In the final CBCT at 36 months, after bar removal, the situation appeared stable, with no implant failures. Treated areas showed further calcification and the formation of a good-quality distal cortical bone.

The obtained results highlight the bone regeneration potential of SmartBone^®^, demonstrating high osteointegration and an effective bone remodeling process in the various analyzed cases. In the treated patients, a progressive mineralization of the grafted material was observed, with increasing integration with the native bone over time. In particular, in cases of different bone defects (mandibular cysts, bone resorption around prosthetic implants, and socket preservation), the formation of a new cortical layer and the increase in bone density were well documented by CBCT images. The results show a good integration capacity in most cases, with a progressive replacement of the grafted material by native bone. Two cases presented challenges related to material stability and proper osteointegration, with instances of fracture or mobility, but only one required surgical revision. After the revision, the patient showed correct osteointegration of the graft. Overall, the collected data confirm the effectiveness of SmartBone^®^ in promoting bone regeneration and implant stability in the long term. The details of each case and their respective results are summarized in Table 1.

### 3.2. Quantification of Remodelling Trend

The use of the xeno-hybrid bone graft material SmartBone^®^ demonstrated a clear trend of bone regeneration, observed through the measurement of ΔCT values over time in various regions of interest. These regions were selected based on their anatomical relevance, ensuring a comprehensive and representative evaluation of the material’s effectiveness. The areas included both the lingual and buccal cortical regions, as well as treated and untreated medullary spaces, to compare bone evolution under different conditions. The lingual and buccal cortical regions were chosen to observe the changes in bone density in areas closest to the graft. These regions are particularly relevant as they represent both the mobile and fixed cortical areas, which respond to bone remodeling processes in a differentiated way. Additionally, the treated and untreated medullary spaces were included in the analysis to study the transformation of the cancellous bone matrix and its adaptation to the implanted xeno-hybrid material. The selection of these specific anatomical areas allowed for a detailed and multifaceted overview of SmartBone^®^’s behavior in relation to osseointegration and bone regeneration processes, highlighting any differences between treated and untreated areas over time.

**Figure 12:**
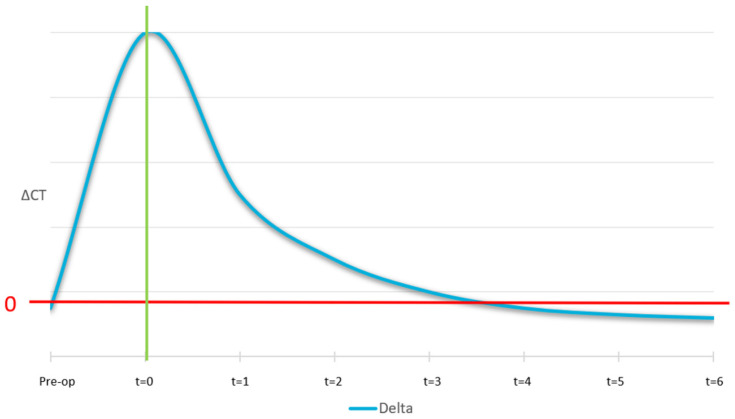
Ideal pattern of Δ mobile cortical vs. time. The curve shows an initial peak at t = 0 due to the presence of non-integrated graft material. Over 4–6 months, ΔCT decreases to zero, indicating successful cortical bone formation.

The ideal curve for the mobile cortical is shown in Figure 12. At the pre-surgical stage, we expect the lingual and buccal cortical to have equal density and, as a result, the ΔCT at t = −1 is zero. At time zero (surgery), the curve shows a peak which is because the graft material is not yet osteointegrated. Then, after 4/6 months, a correct osseointegration induces the formation of a new cortical, with the same intensity as the native one.

**Figure 13:**
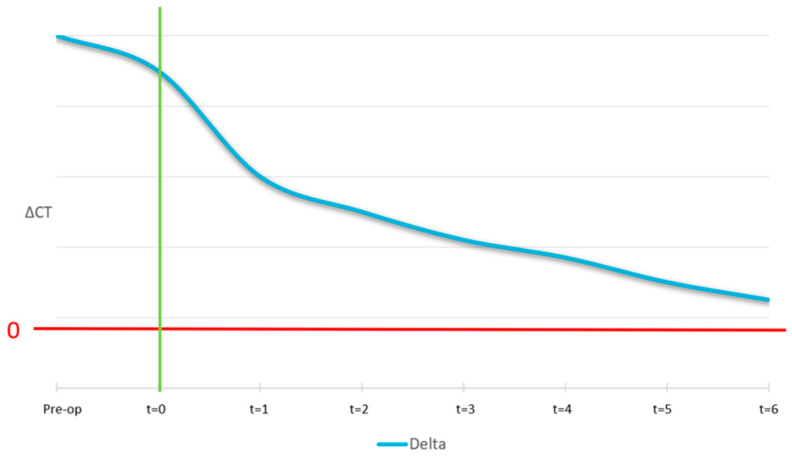
Ideal pattern of Δ medullary vs. time. Initially high ΔCT values decrease over time as the graft remodels into new cancellous bone, achieving density similar to the native medullary region.

The ideal curve for the Δ (delta) medullary is showed in Figure 13. At pre-surgery stage, no new medullary region was available, points of interest were out of bone region, which leads to a higher value of delta medullar with the respect to the medium value. At Time 0 it was not expected the biomaterial to be perfectly thickened, it was therefore considered equal to non-treated. Correct osseointegration induces the formation of a new medium of equal intensity to the non-treated ones.

**Figure 14:**
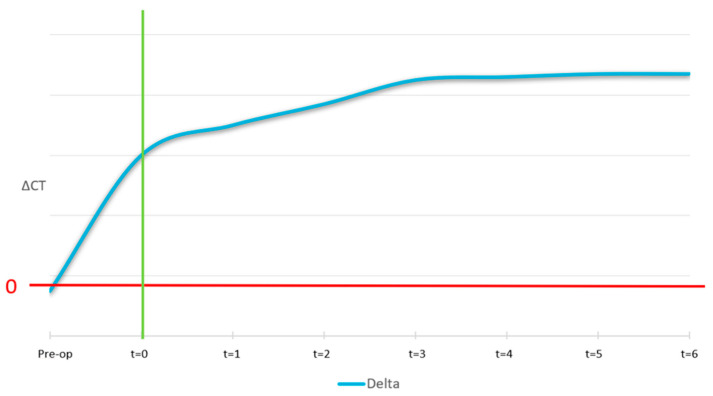
Ideal pattern of Δ fixed cortical vs. time. ΔCT starts at zero, with the original cortical visible at t = 0. Over time, osseointegration leads to its resorption and replacement by new cortical bone.

The ideal pattern of the Δ fixed cortical is shown in Figure 14. Before surgery it was expected the lingual and buccal cortical to have the same bone density. At t = 0 it was expected that the original distal cortical would have been perfectly visible and not yet absorbed. Over time, correct osseointegration induces the resorption of the original cortical in favour of new cortical formation at the outer border of the biomaterial.

The remodelling was quantitatively monitored by comparing the radiology absorbance value at the regions of interest, where remodelling was expected and comparing them to reference locations. This gave a Δ-value that was monitored over time. Three Δ-values were monitored: (A) the difference between the lingual cortical to the buccal surface of the area treated with SmartBone^®^; (B) the cancellous region of the non-treated area to the area where the SmartBone^®^ material was implanted; and (C) the lingual cortical bone to the original buccal cortical.

**Figure 15:**
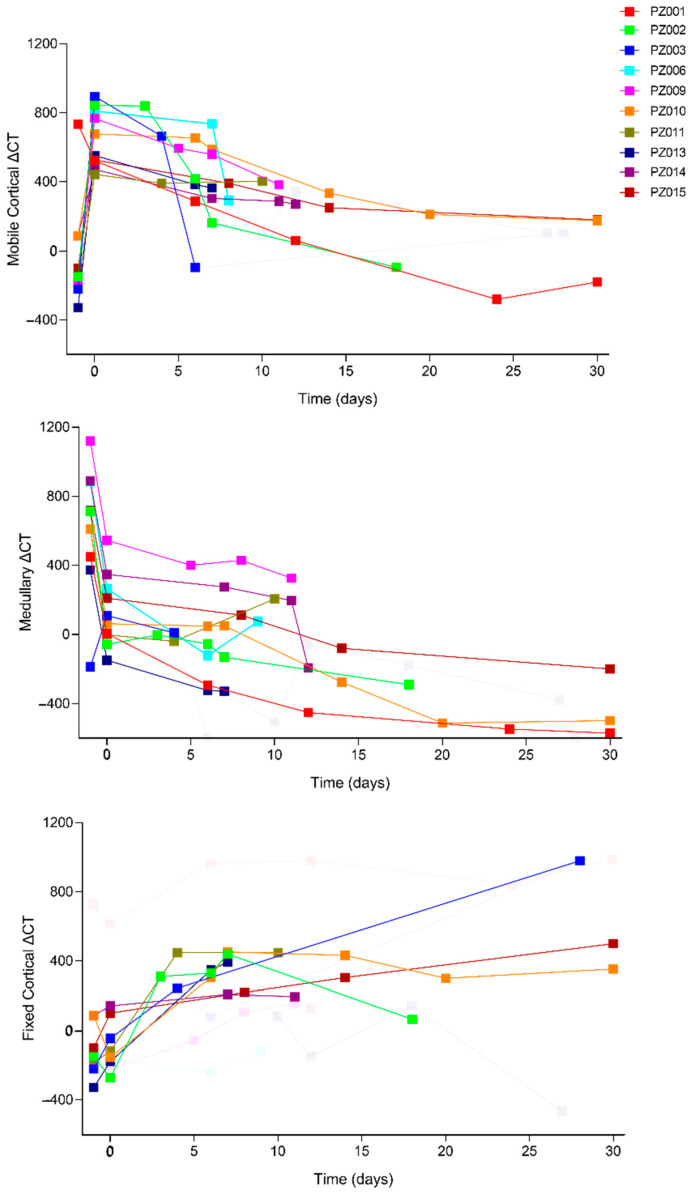
Analysis of bone remodeling over time in patients treated with SmartBone^®^. The trend of ΔCT values in the different regions of interest highlights the process of integration and transformation of the graft into new bone tissue. Panel A represents the mobile cortical ΔCT trend, defined as the difference between the lingual cortical and the buccal cortical surface at the graft site. A decreasing ΔCT indicates progressive integration and remodeling of the graft into new cortical bone. Panel B represents medullary ΔCT trend, showing the difference between CBCT values in the treated medullary region versus an untreated medullary region. The decreasing values over time reflect successful remodeling of the graft into native cancellous bone. Panel C represents fixed cortical ΔCT trend, defined as the difference between the lingual cortical bone and the original buccal cortical bone. The progressive reduction suggests resorption of the original cortical margin and replacement with new bone. Each curve in the panels represents the time-dependent evolution of ΔCT values for individual patients included in the study.

Most importantly, the trend of bone remodeling over time in patients treated with the SmartBone^®^ xenohybrid bone graft is illustrated in Figure 15: the analysis is based on variations in the ΔCT value between different regions of interest (lingual-buccal cortical and treated-untreated medullary), assessing the process of bone integration and remodeling. In panel A, the mobile cortical ΔCT, defined as the difference between the lingual cortical bone and the buccal surface of the grafted area, shows an immediate peak after surgery (Time 0), indicative of the presence of the biomaterial before its osteointegration. Subsequently, a gradual decrease is observed over the following months (between 4 and 6 months), eventually reaching a value close to zero, suggesting the formation of new cortical bone with a density similar to the original one. This indicates that the body was able to remodel the graft margin, transforming it into new cortical bone. Panel B illustrates the medullary ΔCT, which represents the remodeling of the treated medullary region compared to the untreated one. It follows the expected trend, with high values in the initial phases and a progressive reduction toward physiological levels, indicating the transformation of the graft into native bone tissue. Specifically, the medullary ΔCT describes the remodeling of the graft into cancellous bone similar to the native one, demonstrating the effectiveness of the biomaterial in promoting bone regeneration.

Panel C reports the fixed cortical ΔCT, which represents the difference between the lingual cortical bone and the original buccal cortical. Initially, it shows a clear distinction between the native bone and the graft. Over the following months, the original cortical bone is progressively resorbed and replaced by a new cortical structure, suggesting proper integration and bone remodeling. Finally, it was observed that the original cortical bone was resorbed and remodeled into cancellous bone, indicating good graft integration.

In most of the analyzed cases, the observed trend follows the expected model, confirming the effectiveness of SmartBone^®^ in bone regeneration.

The monitoring period varied among patients, as follow-up periods differed for each individual. Additionally, in several cases, late-stage follow-up data were excluded due to artefacts introduced by the placement of dental implants. These results support the effectiveness of SmartBone^®^ in promoting bone remodeling and the formation of new cortical and medullary bone, with timeframes ranging between 6 and 12 months depending on the clinical case.

## 4. Discussion

Osseointegration and bone remodeling are supported by the process of osteoinduction, which is the biological mechanism that induces osteogenesis. This process involves the recruitment of immature cells and their stimulation to differentiate into pre-osteoblasts, contributing to the replacement of the grafted material with new bone tissue. Osteoinduction is a key physiological phenomenon for maintaining bone mass in adults and represents a fundamental aspect of the success of a bone graft. Moreover, for optimal graft integration, it is necessary to create an appropriate “biological chamber,” meaning a receiving site consisting of surrounding vital bone and well protected from the infiltration of connective tissue [2].

This study evaluated the ability of the xeno-hybrid material SmartBone^®^ to promote bone regeneration in a cohort of 10 patients undergoing maxillofacial bone grafting procedures. The 10 cases included different types of surgical interventions. Case 1 involved the treatment of a large mandibular cyst with SmartBone^®^ microchips mixed with growth factors. Case 2 addressed bone resorption around a prosthetic arch using a fencing procedure with SmartBone^®^ microchips and a resorbable collagen membrane. Case 3 focused on socket preservation after the removal of failing dental implants, using SmartBone^®^ microchips and a resorbable collagen membrane. Case 4 targeted bone resorption around a missing tooth, using a SmartBone^®^ plate secured with nails. Case 5 involved the grafting of a thin maxillary bone with deep grooves, using two SmartBone^®^ plates, later followed by implant placement. Case 6 treated the thinning of the left maxillary bone that threatened a dental implant, using a SmartBone^®^ plate secured with nails. Case 7 managed severe bone resorption in the upper jaw by placing a large SmartBone^®^ plate for structural support before implant placement. Case 8 required bilateral SmartBone^®^ On-Demand plates to address bone loss in the upper jaw, allowing for later implant placement. Case 9 dealt with maxillary sinus atrophy, where SmartBone^®^ On-Demand plates were used to restore bone volume before prosthetic rehabilitation. Finally, Case 10 addressed severe bone atrophy in an edentulous upper arch using a grafting procedure with SmartBone^®^ on-demand plates, later followed by implant placement. 

The results of this study confirm the effectiveness of the xeno-hybrid material SmartBone^®^ in promoting bone regeneration in patients undergoing maxillofacial bone grafting procedures. Overall, 9 out of 10 cases were classified as fully successful, one was partially successful without requiring revision (Case 7). In all cases, including the revision, the material demonstrated osteointegrative properties and remodeling ability, suggesting that SmartBone^®^ could represent a valid and effective solution. Bone density analysis using cone beam computed tomography (CBCT) revealed a clear process of bone remodeling, with the progressive resorption of the original distal cortical wall and the formation of a new external cortical wall. This phenomenon was particularly evident in cases of volumetric maxillary bone regeneration, such as in Case 2, where the reconstruction of bone around a prosthetic arch led to the formation of a new cortical layer at the graft margin within six months. In other cases, the remodeling process took longer but ultimately resulted in satisfactory integration of the implanted material with the surrounding bone.

In this clinical study, the material’s ability to support the formation of cortical new bone was demonstrated by comparing bone density values between treated and untreated areas. In general, the difference in density (ΔCT) between the lingual and buccal cortical regions progressively decreased in the months following surgery, indicating proper integration of the material with the native bone structure. Cases were classified as successful when CBCT analysis showed complete graft remodeling with newly formed cortical and medullary bone indistinguishable from native tissue, stable implant placement, and absence of clinical complications. A case was considered partially successful when integration was incomplete or partial resorption of the graft occurred. In many cases, the newly formed cortical bone appeared indistinguishable from the native bone in CBCT images after approximately 12 months, suggesting that the material had been completely resorbed and replaced by regenerated bone. The only partially successful case (Case 7) exhibited partial loss of the grafted material and incomplete osteointegration. However, no surgical revision was required, and the remaining implanted material still contributed to new bone formation, albeit to a lesser extent than in other cases. Follow-up evaluations confirmed that, in all cases, the graft demonstrated osteointegrative properties and the ability to remodel as needed. Remodeling appeared to begin immediately after surgery and continued for a period of 6 to 12 months. The resorption of the original distal cortical wall and the formation of a new distal cortical wall were evident, representing a significant advancement in current surgical strategies for the dental region.

Although the present study does not include a direct comparison with other biomaterials, the literature provides comparative evidence supporting the validity of SmartBone^®^ in regenerative applications. In a clinical study involving patients undergoing bone regeneration, SmartBone^®^ was compared with Bio-Oss^®^, a well-known and widely used xenograft. The results showed that SmartBone^®^ promotes selective bone remodeling, capable of adapting to the specific anatomical requirements of the recipient site, favouring the formation of both cancellous and cortical bone tissue in a functional and predictable manner [25]. Furthermore, in a comparative study with a synthetic calcium phosphosilicate material, SmartBone^®^ demonstrated equally effective clinical and radiological outcomes, especially in sites with significant cortical bone loss, confirming its validity in supporting osteointegration and bone regeneration. These complementary studies confirm SmartBone^®^’s ability not only to support bone regeneration but also to effectively compete with other materials, whether xenografts or synthetics [2].

However, this study presents several limitations. Several factors may have influenced the results, including the small sample size, short/mid-term follow-up times, inter-patient variability in pre-existing bone conditions and the presence of metallic elements in CBCT scans, which could have introduced artefacts. Additionally, the biological response to the graft can be affected by systemic patient factors such as diabetes or vitamin deficiencies, which are not always easily identifiable in clinical data and are often not disclosed to dentists. Moreover, the use of CBCT as the only diagnostic tool poses concerns. As highlighted by Nackaerts et al., CBCT gray values are not reliable for absolute bone density quantification, being influenced by voxel position and metallic artefacts [42]. Therefore, the data obtained should be interpreted as indirect indicators of bone integration and regeneration. The lack of histological confirmation also limits direct evaluation of the type and quality of the new tissue. Despite these limitations, the findings align with previous evidence demonstrating SmartBone^®^’s osteoconductive and regenerative capacity. Histological study conducted by D’Alessandro et al. showed that after four months, SmartBone^®^ coexisted with newly formed bone (43.9%), and by six months, it was almost entirely resorbed (0.5%) and replaced with mature bone (80.8%) [21]. Histological analyses showed well-oriented lamellae, bone scars typical of mature bone, and the presence of bone matrix biomolecules and active osteoblasts. The absence of inflammatory cells and the presence of osteogenic markers confirmed biocompatibility and non-immunogenicity, supporting the conclusion that SmartBone^®^ is osteoconductive and promotes rapid bone regeneration [21]. Another clinical study has demonstrated that SmartBone^®^ supports anatomically selective remodeling: although it is a homogeneous, dense spongy bone graft, it undergoes progressive remodeling that promotes the formation of either cancellous or cortical new bone according to the specific anatomical requirements of the receiving site [2,26,43].

These data support the regenerative trends observed in this study through CBCT analysis and clinical outcomes, suggesting that SmartBone^®^ possesses osteoinductive properties and is capable of integrating and undergoing selective remodeling in vivo in a predictable manner under favourable conditions. However, to improve the quality and robustness of the results, future studies should include quantitative histological and histomorphometric analyses to directly assess the biological behavior of the material over time. Additionally, it would be advisable to enlarge the sample size. Since the current evaluation is based on short- to mid-term follow-up periods, longer-term studies are also needed to better understand the stability, resorption rate, and remodeling dynamics of the graft material in the long run. Nonetheless, a previous study employing computed tomography to assess SmartBone^®^ integration in patients undergoing reconstructive surgery demonstrated new bone formation and volumetric increase within a few months after grafting, indicating effective integration of the material [44]. These findings support the clinical relevance of the short- to mid-term timeframe adopted in the present study.

## 5. Conclusions

This study demonstrates that the xeno-hybrid material SmartBone^®^ effectively supports bone regeneration by promoting selective, site-specific remodeling. The predominant cortical remodeling pattern, with resorption of the original cortical wall and formation of a new external cortical layer facilitates strong integration with surrounding bone, ensuring stability in the treated area. CBCT analysis confirms a gradual equalization of bone density between grafted and native tissue, indicating successful biomaterial replacement by regenerated bone. These results establish SmartBone^®^ as a reliable and promising option for maxillofacial bone grafting. However, the study has some limitations, including the small sample size, mid-term follow-up, inter-patient variability, and the exclusive use of CBCT data without histological validation. While further research is warranted to validate long-term outcomes and overcome these limitations, current evidence positions SmartBone^®^ as a material capable of delivering predictable and stable clinical results.

## Figures and Tables

**Table 1:** Summary of clinical cases. In total, 10 clinical cases are analyzed, detailing the type of bone defect treated, the graft material used, follow-up periods, and key observations on the remodeling process.

	Age	Sex	Defect Type	Graft Type	Follow-up (Months)	Key Findings
1—PZ001	69	M	Large mandibular cyst	Microchips (0.25–1 mm) + Growth Factors	0, 6, 12, 24, 30	Progressive mineralization, formation of new cortical layer at 12 months, full cortical regeneration at 30 months
2—PZ002	54	F	Bone resorption around prosthetic arch	Microchips (0.25–1 mm) + Resorbable membrane	−1, 0, 3, 6, 7, 18	Cortical resorption at 3 months, new cortical wall formed at 6 months, stable maxilla at 18 months
3—PZ003	59	F	Bone resorption between implants (positions 36–35)	Microchips (0.25–1 mm)	−1, 0, 4, 6, 28	Cortical recovery at 4 months, stable cortical structure at 6 months, implants reinserted and stable at 28 months
4—PZ006	59	M	Bone resorption under a bridge (position 43)	Plate (3 × 25 × 15 mm)	−1, 0, 6, 9	Cortical resorption at 6 months, formation of new cortical layer at 9 months
5—PZ009	57	M	Thin maxillary bone, deep grooves	Plates (3 × 25 × 15 mm)	−1, 0, 5, 8, 10	Cortical resorption at 8 months, formation of new cortical layer at 10 months
6—PZ010	44	F	Thinning of left maxilla (implant position 22)	Plate (3 × 25 × 15 mm)	−1, 0, 6, 7, 14, 20, 30	Progressive cortical resorption and remodeling over 30 months, stable cortical integration
7—PZ011	73	F	Bone resorption, multiple devitalized teeth	Plate (3 × 25 × 15 mm)	−1, 0, 4, 10	Partial loss of grafted material, but cortical resorption and remodeling observed
8—PZ013	58	F	Severe bone resorption (bilateral maxilla)	On-Demand	−1, 0, 6, 7, 10	Right side: good cortical integration, left side: incomplete integration, cortical formation only on the right
9—PZ014	63	M	Maxillary sinus atrophy	On-Demand	−1, 0, 7, 11, 14	Initial cortical thinning, new cortical formation at 14 months
10—PZ015	64	F	Sever bone atrophy (edentulous upper arch)	On-Demand	−1, 0, 8, 14, 36	Formation of new distal cortical wall at 8–14 months, stable implants and further calcification at 36 months

## Data Availability

The datasets generated and analyzed during the current study are available from the corresponding author on reasonable request.
